# Duchenne expert physician perspectives on Duchenne newborn screening and early Duchenne care

**DOI:** 10.1002/ajmg.c.31993

**Published:** 2022-08-05

**Authors:** Niki Armstrong, Rachel Schrader, Ryan Fischer, Norah Crossnohere

**Affiliations:** ^1^ Parent Project Muscular Dystrophy Washington District of Columbia USA; ^2^ Present address: College of Medicine, Department of Internal Medicine Division of General Internal Medicine, The Ohio State University Columbus Ohio USA

**Keywords:** Duchenne, newborn screening, provider perspectives

## Abstract

Duchenne muscular dystrophy (DMD) is a progressive, fatal neuromuscular disorder typically diagnosed between 4 and 5 years of age. DMD currently has five FDA approved therapies, which has led to increased interest in newborn screening (NBS) for DMD. Our objective was to explore the perspectives and predicted practices of physicians (primarily neurologists) who will likely be responsible for the follow‐up of infants identified with DMD through NBS. A short survey was developed and distributed to physicians who are responsible for providing care for patients with Duchenne at Certified Duchenne Care Centers across the USA. Twenty‐seven physicians responded to statements about benefit and readiness for dystrophinopathy NBS, which care recommendations they would make at initial infant visits, and when they would recommend initiating approved therapies. Most DMD physicians indicated they see benefit in NBS (82%) and believe the DMD care community is ready for NBS in dystrophinopathies (74%). The majority of physicians would recommend multiple interventions, including genetic counseling, maternal carrier testing, referral to early intervention services, screening siblings, discussion of clinical trials, exon skipping therapies, and assessment of social and language development at initial visits. The majority of physicians also indicated they would recommend initiating approved therapies much earlier than the typical age of diagnosis.

## 1 INTRODUCTION

Duchenne muscular dystrophy (DMD) is a progressive, fatal condition that is typically diagnosed between 4 and 5 years of age (Thomas, 2022). DMD is caused by pathogenic variants in the X‐linked *DMD* gene, resulting in the absence or reduction of dystrophin. DMD and the milder form Becker muscular dystrophy (BMD) are dystrophinopathies that primarily affect boys, with an incidence of approximately 1/5000 live male births (Crisafulli et al., 2020). In DMD, muscle damage accumulates over time, ultimately resulting in muscle cell death and replacement of muscle with fibrosis. Families report noting symptoms in boys with DMD on average 2 years prior to diagnosis (Thomas, 2022). Infants with DMD may appear asymptomatic on generalist exam, but often have delayed early milestones and lower Bayley‐III scores (Connolly, 2013; Gissy et al., 2017).

DMD currently has five FDA‐approved therapies, including a DMD‐specific corticosteroid and four exon‐skipping therapies. The exon skipping therapies are mutation‐specific and currently available for 30% of the DMD population (Bladen et al., 2015). The goal of exon skipping therapies is to produce a truncated but functional dystrophin (Charleston et al., 2018). FDA labels for the exon skipping therapies are age‐agnostic. Treatments that increase the quantity of dystrophin are likely optimized when provided to individuals who have significant remaining muscle tissue and little or no fibrosis (Kharraz, Guerra, Pessina, Serrano, & Muñoz‐Cánoves, 2014). Unfortunately, most boys with DMD are not diagnosed until after they have significant symptoms of muscle weakness, at which time they have already lost muscle tissue and have fibrosis (Vill et al., 2020).

Given our understanding of the pathophysiology of DMD and the development of FDA‐approved therapies, there has been increased interest in newborn screening (NBS) for DMD, with three NBS pilots ongoing in 2021 in the USA (Parad, Sheldon, & Bhattacharjee, 2021; Wynn et al., 2022). Data is being consolidated to support a nomination package for the Recommended Uniform Screening Panel (RUSP), to include DMD as a recommended disorder for NBS across the country. As part of their process to assess NBS for the disease, the RUSP committee also conducts analyses on the preparedness of the NBS community for this disease. This includes both the laboratory as well as the health care providers, primarily neurologists in DMD, expected to participate in follow‐up. Little data exists on the opinions and attitudes towards NBS of the specialists who provide follow‐up for children diagnosed following NBS. There is increased support of DMD experts for NBS (Al‐Zaidy, Lloyd‐Puryear, Kennedy, Lopez, & Mendell, 2017; Chrzanowski, McAnally, & Kang, 2021; Pillers, 2014), but no broader studies evaluating provider opinions have been published.

Because most individuals with DMD are not diagnosed until early childhood, there is also scant data on provider practice for newborns and toddlers. The current care guidelines assume a typical age of diagnosis (Birnkrant, 2018). One expert workgroup did propose clinical follow‐up for DMD NBS, including a potential schedule of visits and referrals including genetic counseling and early intervention (Kwon et al., 2016). Additional efforts are needed to understand provider practice when infants and toddlers are diagnosed. The objective of this study was to explore the perspectives, attitudes, and predicted practices of the physicians likely to be responsible for the follow‐up of infants identified through DMD NBS.

## 2 METHODS

We partnered with an expert working group of health care providers to inform the development of a physician survey on perspectives and predicted practices for follow‐up after DMD NBS. The workgroup was affiliated with a completed NBS pilot (Park, Maloney, Caggana, & Tavakoli, 2022) and composed of neurologists, geneticists, and genetic counselors with considerable experience with DMD and early diagnosis and treatment. The workgroup reviewed draft versions of the survey and provided input on preferred word choice, participant population, response options, and question inclusion.

The final survey included five close‐ended questions, as well as a demographic question regarding specialist type. Survey question and response options are shown in Table 1. General questions on NBS used the term “dystrophinopathy,” as it is known that CK‐MM screening may also detect BMD and/or female carriers (Timonen et al., 2019). Questions regarding initial visits and care used the more specific term “Duchenne,” as DMD, BMD and carrier care all differ. For Question 3, physicians could select multiple options, which were then analyzed in reference to the initial responses regarding benefit of NBS. Question 3 assumed that confirmation of a DMD diagnosis may take up to several months, so the survey respondents were provided with the timeline of less than 6 months. The final survey questions focused on the physician's opinions on optimal ages and recommendations to begin the approved therapies in DMD. Responders were able to skip questions, leaving responses blank.

**TABLE 1:** Survey questions and responses

	Survey question	Response options
1.	I believe there is benefit to newborn screening for dystrophinopathies	Yes
		No
		Uncertain
2.	I believe we are ready as a community who cares for children with dystrophinopathy for newborn screening for dystrophinopathies	Yes
		No
		Uncertain
3.	A baby with a pathogenic variant consistent with a diagnosis of Duchenne is identified as an infant. Which of the following would you recommend at your initial visits (< 6 months of age)? (select all that apply)	Referral to early intervention services for evaluation
		Carrier testing for the mother
		Genetic counseling
		Screening siblings as appropriate, based on age and sex
		Discussion of potential clinical trials
		If eligible, exon skipping therapies
		Discussion of weekend, once weekly or low‐dose corticosteroids
		Assessment of social and language development
4.	A baby eligible for an approved exon‐skipping therapy is identified as an infant. What do you believe is the optimal time to begin exon skipping therapy for maximum benefit?	As soon as possible
		By age 2
		By age 3
		At the age of typical diagnosis, 4–5 years
		I would not prescribe this drug
5.	A baby with a pathogenic variant consistent with a diagnosis of Duchenne is identified as an infant. When do you recommend beginning corticosteroids?	As soon as possible
		By age 2
		By age 3
		At the age of typical diagnosis, 4–5 years

Physician participants were identified through PPMD's Certified Duchenne Care Center (CDCC) program. The CDCC program administers a certification program for neuromuscular centers to demonstrate that they are following the DMD care guidelines (Birnkrant, 2018). The CDCC must have multi‐disciplinary care with expertise in DMD. There were 29 CDCCs at the time of survey administration, with another 24 institutions who have initiated the certification process. Twenty‐eight of the 29 CDCCs follow pediatric patients. Approved CDCCs follow an average of 135 patients with dystrophinopathy per clinic, ensuring that these physicians all have considerable experience with DMD. Recruitment emails were sent to all neurologist and rehabilitation medicine physicians associated with the currently approved and in‐process CDCCs, a total of 81 physicians spanning 53 institutions. The physicians all act as care coordinators for DMD patients and routinely prescribe DMD‐related therapies. The physicians received two emails inviting them to participate.

Participants indicated their consent to participate on the first screen of the electronic survey. No compensation was provided. All study procedures specific to this survey were approved by Geisinger Institutional Review Board (Geisinger Medical Center, Danville, PA, IRB#2022‐0131). No identifying information was collected on the physician participants. Because of the relatively small specialist community, location was considered an identifying characteristic and was not collected.

Physician responses were analyzed descriptively.

## 3 RESULTS

Twenty‐seven physicians (27/81, response rate: 33%) responded to at least one question in the survey. Four participants (15%) identified as rehabilitation medicine physicians and twenty‐three (85%) as neurologists, which is consistent with the CDCC care coordinator mix.

**FIGURE 1:**
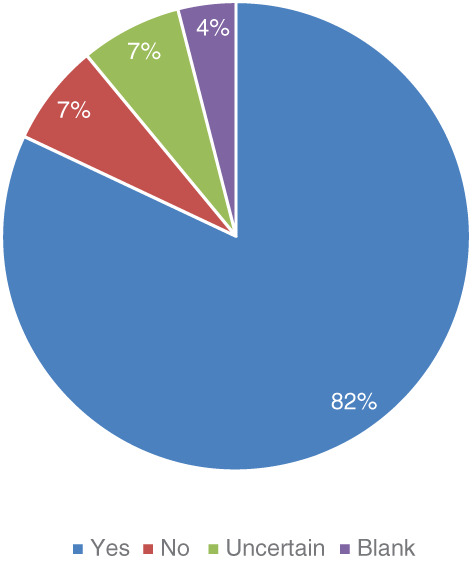
Benefit to newborn screening for dystrophinopathies

**FIGURE 2:**
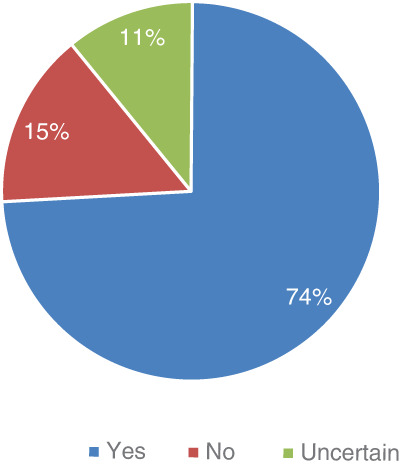
Readiness as a community who cares for children with dystrophinopathy for newborn screening for dystrophinopathies

The vast majority of physicians (82%) indicated that they thought that there was a benefit to conducting NBS for dystrophinopathies (Figure 1). Almost three‐fourths (74%) indicated that DMD care community is ready for NBS for dystrophinopathies (Figure 2).

**FIGURE 3:**
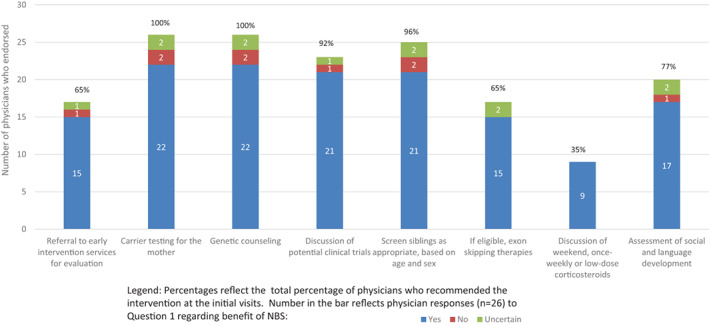
Physician care recommendations for the initial visit. Percentages reflect the total percentage of physicians who recommended the intervention at the initial visits. Number in the bar reflects physician responses (*n* = 26) to Question 1 regarding benefit of NBS

**FIGURE 4:**
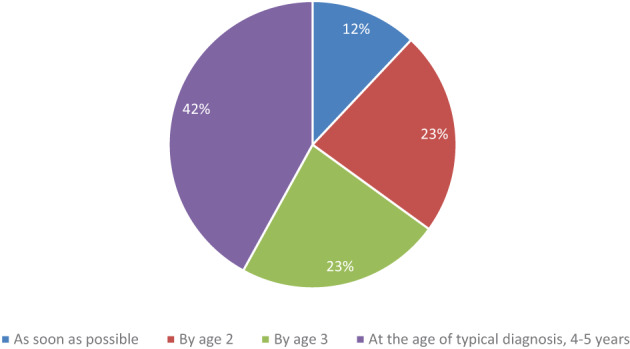
Recommended age to initiate corticosteroids

Regarding the practices of standard DMD care at the initial visits after NBS, all indicated they would recommend genetic counseling and perform carrier testing for the mother. Screening siblings as appropriate based on age and sex (96%), discussion of clinical trials (92%), and assessment of social and language development (77%) were endorsed by the vast majority of physicians (Figure 3). Discussion of corticosteroid therapies was the least endorsed recommendation, selected by only one‐third of physicians (35%). This delay in steroid consideration is further reflected in physician responses to the question regarding when they recommend beginning corticosteroids, as only 12% would begin as soon as possible, with another 23% indicating they recommend prior to age 2 (Figure 4).

**FIGURE 5:**
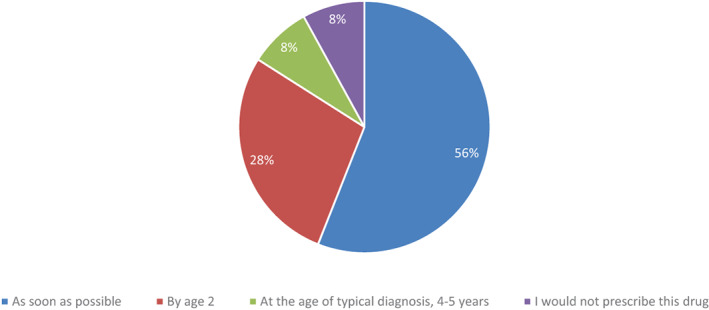
Optimal time to begin exon skipping therapy

More than half of physicians indicated exon skipping therapies should be initiated as soon as possible for optimal benefit, with a large majority (84%) indicating support for initiating exon skipping by age 2 (Figure 5). That said, a small number of physicians (*n* = 2, 8%) indicated they do not prescribe exon skipping therapies.

## 4 DISCUSSION

Primarily due to the typical age of diagnosis, physicians caring for infants and toddlers with DMD have a paucity of data to guide their clinical decision‐making. The majority (84%) still indicated they would start exon skipping therapy prior to age 2, which is more than 2 years prior to the average age of diagnosis of DMD. Current care guidelines do not address age at initiation of exon skipping therapies and limited data exists around treating young patients. Exon skipping therapies received accelerated approval based on clinical trials that were primarily conducted in boys over age 5 who were also on corticosteroids, with a primary endpoint of an increase in dystrophin on muscle biopsy (Lim, Maruyama, & Yokota, 2017). The approved exon skipping therapies have been generally well‐tolerated in older children. Recently released safety data on children ages 6–48 months on eteplirsen suggest they are also well‐tolerated in younger children, although studies have not been conducted on the other exon skipping therapies in this young age group (Mercuri et al., 2022). There is currently no functional or biomarker data on patients treated with exon skipping in the infant or toddler period; however, it is assumed that benefit would occur given that very young boys have more muscle tissue and less fibrosis. Given the positive safety profile, no age limitations on the FDA‐label, and the mechanism of the exon skipping drugs, the high percentage of physicians planning to prescribe exon skipping therapies to young children is understandable.

In this study, a majority (58%) indicated they would recommend corticosteroid initiation prior to age 3, which is significantly younger than the typical age of diagnosis. It is possible that physicians were less likely to recommend steroids before age 2 because of the side effect profile of corticosteroids combined with the lack of efficacy data. Corticosteroids have been well‐studied in older boys with DMD, with improved outcomes in age at loss of ambulation, reduced need for scoliosis surgery, and slowed progression of lung and cardiac disease. While corticosteroids are beneficial in DMD, the side effects of long‐term use are significant and include effects on growth, bone health, and potentially behavioral issues. Current DMD care guidelines recommend a discussion of corticosteroids at the time of diagnosis (Birnkrant, 2018). Studies have shown that the age of initiation of steroids has decreased in recent years, as initiation prior to the plateau period in DMD is optimal (Cowen, Mancini, Martin, Lucas, & Donovan, 2019). A few small studies have looked at infant or toddler initiation, with suggestion of potential benefit, and additional studies are ongoing (Connolly, 2019; Merlini et al., 2012). Deflazacort, the DMD‐specific corticosteroid, is FDA‐labeled for individuals with DMD who are >2 years of age. The mechanism of action of corticosteroids as an anti‐inflammatory is well understood. However, the optimal time to initiate corticosteroids to maximize benefit while minimizing risk is not known. In addition, it may vary depending on steroid type, dosing regimen, and disease progression.

The physician responses regarding when they would recommend therapies are important even in absence of NBS, as many families who have a baby with a potential risk to have DMD based on family history will choose to wait until significant symptoms develop before pursuing diagnostic testing and DMD‐specific care. Ensuring that families and primary care providers are aware that most expert physicians would initiate treatment and assessments prior to the typical age of diagnosis could improve outcomes for these boys.

A minority of physicians (7%) surveyed expressed an opposing viewpoint regarding benefit of NBS for dystrophinopathies. Interestingly, those physicians who indicated they do not see benefit of NBS for dystrophinopathies did still recommend a multiple interventions including carrier testing for the mother, genetic counseling, screening siblings, assessment of social and language development, and referral to early intervention services. Given the significantly increased risk in DMD for language delays and social disabilities including autism, the prompt initiation of these interventions is especially helpful to the boys and their families.

Of the two physicians who indicated they do not see benefit for NBS for dystrophinopathies, one reported not prescribing exon skipping therapies and the other reported that they would not initiate exon skipping until the typical age of diagnosis of 4–5 years. Considering their opinion of not planning to use the approved exon skipping therapies in this early age group, their viewpoint against benefit of NBS can be understood.

Physicians who completed this survey all chose to participate, which may result in a skewing in favor of NBS. It is possible that those who did not complete the survey have different opinions. Response rates are consistent with past research on similar physician specialists' uptake of online surveys provided by an external researcher with no incentive (as an example, neurology physicians in Gilbert et al., 2017). An incentive or longer period of response time may have resulted in a higher response rate and could be considered in additional studies. Neurologists who do not specialize in DMD or who see patients with DMD outside of multi‐disciplinary care centers may have differing opinions.

In the clinic, decisions about therapies are nuanced based on a myriad of factors, including physical exam, family preference, and insurance coverage, which could not be captured in a short survey. Similarly, NBS is a complex system, with multiple potential screening algorithms and approaches. The physicians were asked to respond with very discrete answers to questions that are actually complex. For example, it is possible that some physicians would support DMD NBS only for males or only specifically for the variants that are eligible for exon skipping therapies (Beckers et al., 2021), or that they might consider corticosteroids in a child who is 18 months with severe delays but wait until 3 years of age in a child with more typical development. For the recommendations at initial visits, physicians may have made different choices if the age of the initial visit was more tightly defined or differed; some physicians may make fewer recommendations if the initial visit is less than 3 months of age, for example not referring to early interventions, and more recommendations at an initial visit in an older infant. A survey composed of vignettes, complex questions, or interviews could be considered to better describe the nuances of care for these young ages.

In the past, there have been few clinical trials or even natural history studies on infants and toddlers with Duchenne. Fortunately, a number of studies enrolling infants and toddlers with DMD are currently ongoing, in analysis, or planned to recruit over the next year. While these studies and trials are critically important, many have struggled to enroll participants. Early diagnoses achieved from NBS would allow families to consider these early clinical trials.

The current DMD care considerations were published in 2018, soon after the approval of the first exon skipping therapy. They do not discuss when exon skipping therapies should be initiated. In addition, their discussion of corticosteroid initiation is limited to “at the time of diagnosis,” with a presumed assumption of a typical age of diagnosis. Given the rapid advancements in therapies, updating the considerations to better address these topics or at least to provide a guideline for when to consider would be very useful to providers. Even the FDA‐approved exon skipping therapies may be difficult to access for those less than 4 years. Many insurance companies have developed prior authorization guidelines that limit the therapies to those within the initial trials. Without published efficacy data or care guidelines expressly mentioning younger boys, physicians may struggle to obtain these therapies.

Future considerations include establishing a consortium of experts to further discuss infant and toddler Duchenne care. It is also important to understand how general pediatrician perspectives may vary from those of Duchenne specialists.

## 5 CONCLUSIONS

Overall, the survey responses suggest that expert DMD care physicians see benefit in NBS for dystrophinopathies and believe the DMD care community is ready for NBS for dystrophinopathies. Most physicians surveyed would recommend multiple interventions, including genetic counseling, maternal carrier testing, referral to early intervention services, screening siblings, discussion of clinical trials, exon skipping therapies, and assessment of social and language development at initial visits in infancy. In addition, the majority of expert physicians indicated they would initiate approved therapies much earlier than the typical age of diagnosis if they had that option.

## CONFLICT OF INTEREST

None.

## Data Availability

The data that support the findings of this study are available from the corresponding author upon reasonable request.
